# A Case of Syrian Child with Cerebral Infarction as an Extraintestinal Manifestation of Ulcerative Colitis

**DOI:** 10.1155/2019/5984094

**Published:** 2019-02-26

**Authors:** Riham Salloum, Nawras Alhalabi, Mohamad Anas Almidani

**Affiliations:** ^1^Faculty of Medicine, Syrian Private University, Damascus, Syria; ^2^Department of Neurology, Damascus Hospital, Damascus, Syria

## Abstract

Thromboembolic complications are rare but well-recognized manifestation of ulcerative colitis, especially because of their associated high mortality. We report a case of a Syrian child admitted to Damascus Hospital with a one-day complaint of sudden onset of numbness followed by weakness in the left lower and upper limbs, right mouth angle deviation, and loss of sphincters' control. Earlier, she was diagnosed with ulcerative colitis and treated with immunosuppressants. CT and MRI scans revealed focal infarction around the M2-M3 segments of the right middle cerebral artery; she was treated with Aspirin. On discharge, she had significant improved neurological examination and was able to walk. Subsequent proctocolectomy was performed. We highlight the importance of thromboembolism in ulcerative colitis as there is paucity in the literature regarding its management and its symptoms may be overlooked especially in high-load central hospitals. We conducted a brief literature search and summarized findings of similar reported cases.

## 1. Introduction

Extraintestinal manifestations of idiopathic inflammatory bowel disease (IBD) have been reported in 25% to 36% of patients [1]. Common manifestations include sacroiliitis (14%) and peripheral arthritis (10.7%), while rare manifestations include ocular (8%), mucocutaneous (2.7%), and vascular (2%) [2].

Neurologic manifestations in IBD appear to be more common than previously estimated with a reported incidence of cerebrovascular complications in 0.12% to 4% of all patients with IBD [3, 4]. Generally, it occurs as a postoperative complication and found more in Crohn's disease than ulcerative colitis (UC) [5]. Thromboembolic complications of UC are reported at an incidence of only 1.2%-7.5%, but are well recognized because of their associated high mortality [6–9] which occurs in 60% of cases [4].

We report a rare case of a Syrian child who was suffered a cerebrovascular accident (CVA) as a complication of ulcerative colitis. To the best of our knowledge, this is the first documented case in Syria.

## 2. Case Presentation

A 15-year-old Syrian female was admitted to the hospital on November 2016 with a one-day complaint of sudden onset of numbness in the left lower and upper limbs, followed by weakness in the same areas, right mouth angle deviation, and loss of sphincters' control. She did not experience headache, nausea, vomiting, convulsions, or coma.

Eight months earlier, she developed massive rectal bleeding, colonoscopy was performed, and the patient was diagnosed with ulcerative colitis (UC). She was treated with mesalazine 1 gram three times daily, azathioprine 50 milligram daily, prednisolone 40 milligram daily, and cefuroxime 500 milligram tab twice daily for a week.

She has no history of smoking, alcohol abuse, or illicit drug use. She did not report any suspected allergies and she has no other history of hypertension, diabetes mellitus, cardiac, rheumatological, or hematological disease.

On examination, her vital signs are blood pressure 100/60 mmhg, Pulse 110/minute, respiratory rate 36/minute, and temperature 37.5°C. General examination revealed conjunctival pallor and pitting edema in the left lower limb and purple stretch marks extends on the whole lower limbs till the sacrum.

On neurological examination, there was no impaired consciousness and the patient was awake and alert. Cranial nerves exam was only significant for left facial nerve palsy. Motor examination showed 5/5 strength in the right upper and lower limbs, 3/5 left upper limb, and 0/5 left lower limb; there was also hypotonia on the left limbs and normal tone on the right limbs without any atrophy. Reflexes examinations scored 2/4 for the right limbs (normal) and 1/4 for the left limbs (hyporeflexia). Right toes showed planter flexion and absence of the flexion for the left toes. No cerebellar abnormalities were noted in the right side; cerebellar exam was not performed on the left side due to limbs weakness. She scored 10 on National Institutes of Health Stroke Scale (NIHSS). Sensory examination revealed loss superficial and deep sensations on the left side and normal sensations on the right side. Other systematic examinations, including cardiac, respiratory, and gastrointestinal systems, were all normal.

Investigations including blood tests showed evidence of pancytopenia (hemoglobin 4.4 g/dL, platelets: 66 x1000/mm3 dropped to 3 x1000/mm3 after in two days of admission, WBCs: 1.4 x1000/mm3 with 35% neutrocytes, 61% lymphocytes, 3% monocytes, and 1% eosinophils); urinalysis values were within normal ranges. Thrombophilic and immunological screening including homocysteine, factor V Leiden, protein C, protein S, antithrombin, lupus anticoagulant factor, and antiphospholipid antibodies were all insignificant.

**Figure 1:**
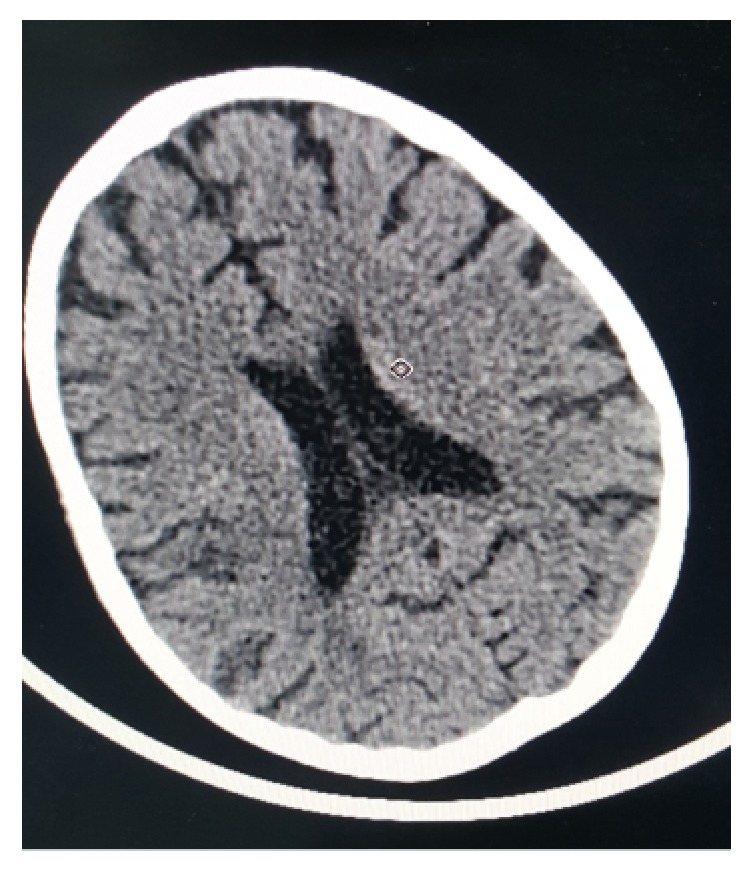
Patient CT scan showing showed small hypodensity foci situated in the cortical and subcortical area in right partial lobe.

**Figure 2:**
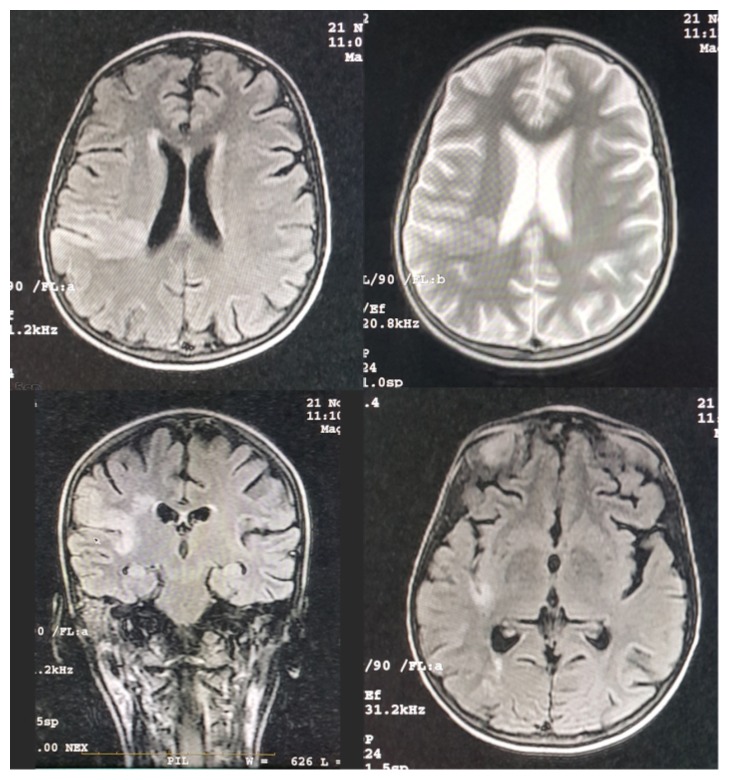
Patient MRI showing cortical and subcortical areas in the right temporoparietal fossa with high signal on T2 and FALIR studies, in addition to isointense foci in the cortical and subcortical area in the posterior part of the parietal lobe extending deeply through the posterior horn of the right lateral ventricle on T1 study.

An emergency computerized tomography (CT) scan (Figure 1) showed small hypodensity foci situated in the cortical and subcortical area in right partial lobe. Magnetic resonance imaging (MRI) (Figure 2) showed cortical and subcortical areas in the right temporoparietal fossa with high signal on T2 and FALIR studies. T1 study showed isointense foci in the cortical and subcortical area in the posterior part of the parietal lobe extending deeply through the posterior horn of the right lateral ventricle. Based on these findings, the accident is complicated with focal infarction around the M2-M3 segments of the right middle cerebral artery. Aspirin 162mg was given upon these findings and the prednisolone treatment was continued.

Cardiac echocardiogram and carotid arteries Doppler ultrasound study were both normal. The patient did not complain of any symptoms related to her UC when she had the CVA, which indicates that the UC was not in active stage.

On December 2016, the patient was able to walk and her neurological examination dramatically improved (NIHSS: 0); she was then discharged and referred to physical therapy. On January 2017, the patient suffered from overt rectal bleeding, she was admitted again to the hospital, and proctocolectomy was performed.

A written informed consent was obtained from the patient before writing this report, Syrian Private University and Damascus Hospital Ethical Committee approved the report, and both are available upon request.

## 3. Discussion

Increased coagulability and thrombosis due to IBD were first described in 1936 [29]. Intestinal inflammation may lead to increased risk for thrombosis through several pathways: activation of coagulation cascade, decreasing anticoagulant activity, inducing hypofibrinolysis, malabsorption, and hypercatabolism with vitamin deficiencies [4]. Most patients with IBD do not have demonstrable specific coagulation defects [30]. Dehydration, immobility, sepsis, surgery, and corticosteroid therapy are also risk factor for thrombosis in IBD patients [31]. The precise mechanism of these factors remains unclear. Arterial thrombosis particularly strokes may be considered a rare condition [17] but with high morbidity and mortality [32–34].

Males and females may be equally affected which correlates with previously reported cases. The cerebral vascular involvement seems more frequent among younger IBD patients [4]. Conventional CT scan or MRI is used to define the cerebral affected areas. At this moment, no guidelines are available for the treatment of cerebral thrombosis and strokes in IBD [17].

By reviewing previous literature, Schneiderman et al. were the first to report similar case with thrombosis of the left internal carotid artery (ICA) and occlusion of the left distal basilar artery in two separate patients with UC respectively [26]. The youngest patient reported was a 1-year-old girl with UC, who was complicated with bacterial endocarditis and subsequent infarctions of both middle cerebral arteries (MCA) [27].

We have conducted a brief literature search and summarized cases reported on Cerebral Arterial Thrombosis associated with UC using modified version of Katsanos et al. [35] table (Table 1).

About half of the patients were on corticosteroid treatment and more than one-third of them were being treated with 5-aminosalicylic acid (5-ASA) at the time of the cerebrovascular event [35]. The predominant neurological symptom on admission in most of the case reports was left or right sided hemiparesis; our patient was also admitted with left hemiparesis as the main complication. In addition, the right or left MCA was the most frequent sites of cerebral arterial thromboembolism depicted on imaging studies similar to our patient that manifested with cerebral arterial thromboembolism around the M2-M3 segments of the right MCA as confirmed by imaging studies [35]. As previously reported, the risk of arterial thromboembolic events may be increased in patients with active disease [4], although our patient did not have an active disease by the time of CVA.

Thrombocytosis and anemia were the most commonly observed potential risk factors for cerebral arterial infarction in the laboratory analysis [35], although there is no sufficient evidence supporting the theory of solitary thrombocytosis causing thromboembolic phenomena [26]. Our patient contrary presented with pancytopenia which may be due to immunosuppressants given [36].

Our patient did not have a demonstrable specific coagulation defects, in comparison; hyperhomocysteinemia [4] and other acquired deficiencies of antithrombin III and protein S have previously been reported in similar cases [30, 35]. Finally, smoking and severe dehydration have been mentioned in only two cases contrary to our patient.

Our patient received Aspirin as a treatment after she was diagnosed with the cerebrovascular accident. She had improved neurological examination on discharge and she was able to walk; she was then discharged and referred to physical therapy, although as reported in previous cases also most of the patients also recovered either without any or with minor neurological deficits; either they received anticoagulation or antiplatelet treatment or not [35] (Table 1).

Clinical experience treatment of arterial ischemic cerebral lesions in patients with IBD is very limited due to lack of enough trials [37]. More studies are required to clarify the correlation between IBD and the thrombophilias and to evaluate the role of anticoagulant therapy and proctocolectomy in the management of these patients. Similarly, there is also lack of steady evidence and official guidelines for stroke management in both children and adults with IBD comorbidity. Both American Heart Association and European Stroke Organization guidelines for stroke management and prevention in the general population are currently presented as a reference point for the treatment of IBD patients who are complicated by an ischemic cerebral event [38–40].

## Figures and Tables

**Table 1:** Cases reported in the international of Cerebral Arterial Thrombosis associated with ulcerative colitis.

Author	Date	Sex	Age	UC Activity	IBD Treatment	Neurological symptoms	Site of cerebral injury/neurological findings	Risk Factor	Thrombophilia screening	Anticoagulation/antiplatelet treatment	Follow-up/outcome
Calabro et al. [10]	2011	F	49	No		Right hemiparesis, aphasia, altered conscious	Left frontotemporal infarct, lesion in the left mesencephalic area, left ICA stenosis	Hypercholesterolemia, elevated Lpa		LMWH (nadroparine 0.6 ml/d), aspirin (300 mg/d) / clopidogrel (75 mg/d)	Improved
Casella et al. [11]	2013	M	25	Yes		Left hemiparesis, confusion, sensitive impairment, bladder incontinence	Right MCA infarct	Anemia		Local thrombolysis/ LMWH and aspirin (100 mg/d)	Complete recovery
Chetri et al. [12]	2002	M	42	Yes	Steroids, 5-ASA	Left hemiplegia	Right MCA infarct and right CCA occlusion	Anemia			Improved
Fukuhara et al. [13]	1993	M	18	No	Steroids	Left hemiparesis	Right paramedian branch of the basilar artery infarct, Ventromedial pons	Anemia			Complete recovery
Hilton-Jones et al. [14]	1985	M	20	No			Basal ganglia infarct	Anemia, abnormal platelet aggregation			Recurrent thrombotic episodes, death
Houissa et al. [4]	2011	M	24	Yes	Steroids	Left hemiplegia	Left MCA infarct		Neg	LMWH	Complete recovery
Houissa et al. [4]	2011	F	25	Yes	5-ASA	Left hemiparesis	Tenticular & right thalamic infarcts	Thrombocytosis	Neg	LMWH	Partial recovery (Residual right hemiparesis)
Jorens et al. [15]	1990	M	31	Yes	5-ASA	Right hemiparesis, nonconfluent aphasia	Recent ischemic lesion in left internal capsule and old ischemic lesion in left basal ganglia	Anemia, thrombocytosis, pro-C, pro-S and prothrombin deficiency, history of TIA			Partial recovery
Jorens et al. [16]	1991	M	32	Yes		Left hemiparesis, stupor, hemiataxia	Temporoparietal cerebrovascular ischemic lesion				Complete recovery
Joshi et al. [17]	2008	M	55	Yes	Steroids, 5-ASA	Left hemiparesis	Right parietal lobe infarction	Thrombocytosis	Neg	LMWH	Partial recovery
Joshi et al. [17]	2008	M	24	Yes	Steroids, 5-ASA	Headache, altered conscious, global aphasia	Left MCA infarct	Thrombocytosis	Neg		Complete recovery, subsequent epilepsy
Keene et al. [18]	2001	F	13	Yes	Steroids, subtotal colectomy	Seizure	Multiple bilateral cerebellar and corona radiata infarcts	Anemia			Complete recovery
Kelly et al. [19]	2014	M	38	No	Steroids, AZA, infliximab	bilateral lower limb claudication, acute confusion with associated ataxia and diplopia	right medial temporal lobe extending posterior to the occipital lobe		Neg	Warfarin	Controlled
Lloyd-Still and To masi [20]	1989	M	5	Yes	Steroids, 5-ASA	Right hemiparesis, seizure	Left MCA arteritis	Anemia			Partial recovery, epilepsy developed 10 years later
Mayeux and Fahn [21]	1978	M	17	Yes	Steroids		Right posterior frontal area infarction				Slowly improved
Nogami et al. [22]	2007	F	26	Yes	Steroids	Left hemiparesis	Right MCA Infarct and right CCA occlusion	Severe anemia		Heparin (15.000 U/d)	Massive GI bleeding, no improvement
Paradis et al. [23]	1985	F	12	Yes		Right hemiparesis, seizure	Left major cerebral vessels occlusion	Anemia, thrombocytosis			Complete recovery
Patterson et al. [24]	1971	M	11	Yes			Cerebral emboli				Colectomy
Richard et al. [25]	2014	F	42	Yes	Steroids, AZA	Sudden right hemiplegia	Right MCA	Slight hyperhomocysteinemia		LMWH, Enoxaparin sodium, Aspirin	
Salloum et al. (our case)	2016	F	15	No	Steroids, 5-ASA, AZA	Left hemiparesis, 7th CN palsy	M2-M3 segments of the Right MCA	Pancytopenia	Neg	Aspirin 162mg	Colectomy, complete recovery
Schneiderman et al. [26]	1979	M	34	Yes		Right hemiplegia, nonfluent aphasia	Left ICA thrombosis	Thrombocytosis		Thrombectomy, heparin/warfarin	
Schneiderman et al. [26]	1979	F	12	Yes	Steroids, 5-ASA	Hemianopia, headache, seizures	Distal basilar artery defect extending to the left PCA	Elevated fVIII			Death
Tomomasa et al. [27]	1993	F	1	Yes	Steroids, 5-ASA	Right hemiplegia, altered conscious, seizures	Left anterior & MCA infarct/ right MCA infarct	Thrombocytosis, bacterial endocarditis			No improvement
Yassinger et al. [28]	1976	F	15	Yes	Steroids		Right frontal lobe infarction				Recovered

M = male; F = female; 5-ASA = 5-aminosalicylic acid; AZA = azathioprine; CN = cranial nerve; ICA = internal carotid artery; MCA = middle cerebral artery; CCA = common carotid artery; PCA = posterior cerebral artery; LPa = lipoprotein; Pro-C = protein C; Pro-S = protein S; TIA = transient ischemic attack; fVIII = factor VIII; LMWH = low molecular weight heparin; GI = gastrointestinal.
